# Identifying Asian American lung cancer disparities: A novel analytic approach

**DOI:** 10.1016/j.xjon.2024.04.010

**Published:** 2024-04-22

**Authors:** Yunna Gu, Les R. Becker, Puja G. Khaitan, John F. Lazar

**Affiliations:** a Georgetown University School of Medicine, Washington, DC; b MedStar Institute for Innovation, Simulation Training, and Education Lab, MedStar Health, Washington, DC; c Department of Thoracic Surgery, Sheikh Shakhbout Medical City, Abu Dhabi, United Arab Emirates; d Division of Thoracic Surgery, MedStar Washington Hospital Center, Washington, DC

**Keywords:** health care disparities, lung cancer, smoking cessation, Asian Americans, columnar *z*-score analysis, lung cancer survivorship

## Abstract

**Objective:**

Asian Americans include heterogeneous subpopulations with unique burden as the only racial group with cancer as the leading cause of death. The purpose of the study was to identify differences in clinical stage and survival of patients with lung cancer between Asian Americans and its subgroups relative to other racial groups.

**Methods:**

Patients with lung cancer from 2016 National Cancer Database were divided into East Asian, Southeast Asian, South Asian subgroups based on geographic origins, and a composite Asian American group with White non-Hispanic, Black, and Hispanic comparison groups. Columnar *z* score analysis with adjusted residuals was employed and the terms underrepresented and overrepresented were utilized to describe significant statistical findings.

**Results:**

A total of 825,448 patients were analyzed. Asian Americans were underrepresented relative to White non-Hispanics in all clinical stages except IIIB and IV. In clinical stage IV, Asian Americans (51.0%), East Asians (47.2%), Southeast Asians (57.4%), and South Asians (52.2%) were overrepresented relative to White non-Hispanics (42.2%) and Southeast Asians were overrepresented relative to East Asians and South Asians. For survival across all stages, Asian Americans were overrepresented relative to White non-Hispanics and Blacks, but in clinical stage IV, Southeast Asians (17.9%) were underrepresented relative to East Asians (26.0%) and South Asians (26.6%).

**Conclusions:**

This is the first study to address lung cancer disparity in Asian American subgroups employing a novel analytical approach. Asian American subgroups demonstrated more advanced lung cancer diagnosis yet higher survival compared with White non-Hispanics, Blacks, and/or Hispanics with differences between subgroups. Interplay of complex factors may contribute to Asian American health disparities.

## Graphical Abstract

**Figure:**
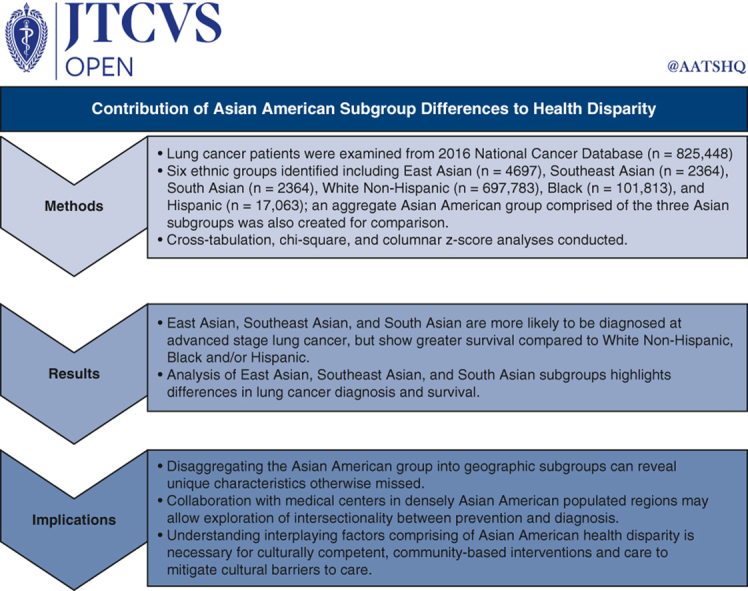

**Figure:**
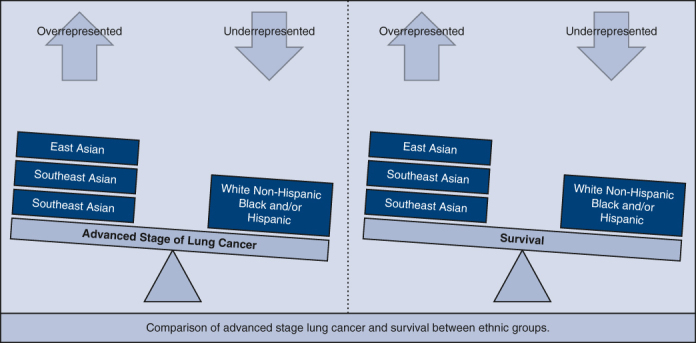
East Asian, Southeast Asian, South Asian are more likely to be diagnosed at advanced stages of lung cancer yet demonstrate higher survival compared to White Non-Hispanic, Black, and/or Hispanic with differences between subgroups.

**Central Message**

Asian American subgroups are more likely to be diagnosed with advanced lung cancer yet show greater survival compared with White non-Hispanics, Blacks, and/or Hispanics with differences between subgroups.

**Perspective**

First study addressing lung cancer disparity in Asian American subgroups defined by geographic origins with a novel analytical approach. Asian Americans are more likely to be diagnosed with advanced lung cancer yet greater survival compared with White Non-Hispanics, Blacks, and/or Hispanics with differences between subgroups. Interplay of complex factors may contribute to health disparities.

See Discussion on page 165.

Asian is defined as “a person having origins in any of the original peoples of the Far East, Southeast Asia, or the Indian subcontinent” by the US Census Bureau.^1^ In the 2020 US Census, Asian, Native Hawaiian, or Other Pacific Islanders alone accounted for 6.2% of the total US population, which increased by 35.6% since 2010 compared with a 7.4% increase in the total US population.^2^, ^3^, ^4^ In addition, Asian Americans have been the fastest growing ethnic group in the past decades in the United States and are projected to increase by 79% by 2050.^5^ Similar to Hispanic or Latino, Asian American is an umbrella term that covers 21 different peoples of origin with distinct culture, language, history, religion, political systems, demographics, and socioeconomic backgrounds. Due to the heterogeneous nature of the Asian American population, generalizing any 1 health care issue has been a significant challenge.

For Asian Americans, lung cancer is perhaps the single greatest health burden because they are the only racial group experiencing cancer as the leading cause of death for both women and men at 25.5% and 24.8%, respectively. This is in stark contrast to all other US racial groups, besides Hispanic women, where heart disease is the leading cause of death.^6^ From 1990 to 2008, lung cancer consistently ranked among the top-4 cancer sites in Asian Americans with increasing trends among South Asian men, Filipinas, and Korean women. Lung cancer was the most common neoplasm in Kampuchean, Laotian, and Vietnamese men and second most common neoplasm in Indians, Pakistanis, Chinese, Filipinos, Japanese, and Koreans.^7^ From 1972 to 1988, Southeast Asian men had 18% higher incidence of lung cancer than Whites in Los Angeles County.^8^

Endemic to most immigrants in the United States is the existence of health disparities. Factors contributing to the prevalence of lung cancer and health disparities for Asian Americans are multifaceted and include the influence of the subgroup's home culture and its integration with US culture, which is extremely nuanced. This can be attributed to a complex interplay of factors such as socioeconomic status, access to health care, language barriers, level of education, cultural beliefs, and discrimination.^9^, ^10^, ^11^, ^12^, ^13^

In the past, a major obstacle to studying lung cancer in Asian Americans has been the small sample size. The chief bias associated with this approach is that by aggregating Asian American data, it masks valuable identifiable characteristics between ethnic subgroups, especially in evaluating health disparities.^10^ To overcome this limitation, a columnar *z*-score analysis with adjusted standardized residuals was applied to Asian American subgroups identified by geographic origins and the terms *underrepresented* and *overrepresented* were utilized to describe significant statistical findings. To our knowledge, this approach was novel to health disparity studies. The primary end point was to identify differences in clinical stage at diagnosis and survival of patients with lung cancer between Asian American subgroups and other racial groups.

## Methods

Patients with lung cancer, identified by International Classification of Diseases for Oncology, Third Revision, code C34, were extracted from the 2016 National Cancer Database (NCDB), which included both small cell and non–small cell lung cancer (NSCLC) from 2004 to 2016. The American Joint Committee on Cancer Cancer Staging Manual, sixth edition, was used for cases diagnosed from 2004 to 2009 and the seventh edition was used for cases diagnosed from 2010 to 2016. The Medstar Health Research Institutional Review Board approved the study protocol and publication of data. The informed written consent of patient(s) for the publication of the study data was not required because the NCDB provides de-identified data (institutional review board STUDY00000285, approved March 3, 2019). Cases characterized by American Joint Committee on Cancer clinical stage IA, IB, IIA, IIB, IIIA, IIIB, and IV were retained for further analysis. A significant limitation in prior studies examining lung cancer disparities in Asian Americans is the relatively small proportion of patients who identify as Asian American. To combat this limitation as well as ensure statistical significance and generalizability, 11 Asian American ethnicities were divided into 3 Asian American subgroups based on geographic origins. In addition, we pooled the 3 subgroups to create an Asian American comparison group. A total of 6 ethnic groups were identified in this study as White non-Hispanic, Black, Hispanic, East Asian, Southeast Asian, and South Asian. East Asians were defined as Chinese, Japanese, and Korean. Southeast Asians were defined as Filipino, Vietnamese, Laotian, Hmong, and Thai. South Asians were defined as Indian, Pakistani, and Asian/Indian/Pakistani not otherwise specified. Patients were excluded if clinical stage was 0, 0A, 0is, occult, or unknown and if race was other or unknown. Cross-tabulation analyses were conducted to explore distributional differences across demographic and clinical variables (SPSS version 28; IBM-SPSS Inc). Columnar *z*-score analysis with Bonferroni correction was employed to identify statistically significant differences between all possible paired comparisons in a table row.^14^, ^15^, ^16^ The χ^2^ values were calculated to assess each cross-tabulation table. This cross-tabulation approach compares each row entry with every other row entry, assigning an incremented subscript to each row entry as a significant columnar difference is identified. Row entries with common subscript letters designate ethnic group categories whose column proportions; that is, the row entry, do not differ significantly from each other at the .05 level. Conversely, row entries with different subscript letters indicate row entries that do differ significantly from each other at the .05 level. The magnitude and directionality of the adjusted residual was generated for each table cell. The terms *overrepresented* and *underrepresented* were employed to describe significant *z-*score findings at *P* < .05 with an adjusted residual > +2 or < −2 indicating over- and underrepresentation, respectively. Where appropriate, the magnitude of similarly assigned residuals were compared to assign relative over- and underrepresentation. Agresti^16^ supports the interpretation of “sample percentages; ” that is, our column percentages and residual in this fashion.

## Results

### Demographics and Socioeconomic Factors

A total of 825,448 patients were included, resulting in a cohort that was 84.5% White non-Hispanic, 12.3% Black, 2.1% Hispanic, 0.6% East Asian, 0.3% Southeast Asian, and 0.2% South Asian. The proportions of male and female patients were similar within each ethnic group (Table 1). East Asians, Southeast Asians, and South Asians were underrepresented in the first median income quartile and overrepresented in the fourth median income quartile relative to White non-Hispanics, Blacks, and Hispanics (Table 2). East Asians, Southeast Asians, and South Asians were overrepresented as not insured and Medicaid primary payers and underrepresented as Medicare primary payers relative to White non-Hispanics. Southeast Asians and South Asians were more likely to be private insurance/managed care payers relative to all other ethnic subgroups (Table 3). East Asians and South Asians were overrepresented in the first quartile of noncompletion of high school relative to White non-Hispanics, Blacks, and Hispanics, whereas East Asians were also overrepresented relative to White non-Hispanics in the fourth quartile (Table 4).

**Table 1:** Demographic breakdown

Ethnic group	Sex				Total	
	Male		Female			
WNH	368,721	(84.1)	329,042	(85.1)	697,763	(84.5)
Black	55,267	(12.6)	46,546	(12.0)	101,813	(12.3)
Hispanic	9830	(2.2)	7233	(1.9)	17,063	(2.1)
EA	2475	(0.6)	2222	(0.6)	4697	(0.6)
SEA	1282	(0.3)	1082	(0.3)	2364	(0.3)
SA	1083	(0.2)	665	(0.2)	1748	(0.2)
Total	438,658	(100.0)	386,790	(100.0)	825,448	(100.0)

Values are presented as n (%). *WNH*, White Non-Hispanic; *EA*, East Asian (Chinese, Japanese, Korean); *SEA*, Southeast Asian (Filipino, Vietnamese, Laotian, Hmong, Thai); *SA*, South Asian (Indian, Pakistani, Asian Indian/Pakistani not otherwise specified).

**Table 2:** Median income quartile × ethnic group cross-tabulation

Median income	Ethnic group						Total
	WNH	Black	Hispanic	EA	SEA	SA	
First quartile∗ <$40,227							
Count	128,208_a_	53,277_b_	5236_c_	687_d_	255_e_	151_e_	18,7814
% within ethnic group	18.6	53.1	31.0	14.7	10.9	8.7	23.1
Adjusted residual	−222.1	241.1	24.7	−13.6	−14.1	−14.2	
Fourth quartile∗ ≥$63,333							
Count	229,561_a_	13,932_b_	4074_c_	2220_d_	1121_d_	1011_e_	251,919
% within ethnic group	33.3	13.9	24.1	47.5	47.7	58.3	30.9
Adjusted residual	110.2	−124.9	−19.4	24.6	17.7	24.7	

*WNH*, White non-Hispanic; *EA*, East Asian (Chinese, Japanese, Korean); *SEA*, Southeast Asian (Filipino, Vietnamese, Laotian, Hmong, Thai); *SA*, South Asian (Indian, Pakistani, Asian Indian/Pakistani not otherwise specified).

∗ Within a given table row, cells annotated with the same subscript letter indicate that the column proportions do not differ significantly from each other at the .05 level. Similarly, within a given table row, cells annotated with different subscript letters indicate that the column proportions do differ significantly from each other at the .05 level. For example, for the First Quartile row: every ethnic group is significantly different from each other execpt for SEA and SA, who are not significantly different from each other.

**Table 3:** Primary insurance payer × ethnic group cross-tabulation

Insurance status∗	Ethnic group						Total
	WNH	Black	Hispanic	EA	SEA	SA	
Not insured							
Count	17,218_a_	5351_b_	1019_c_	190_d_	168_c_	119_b,c_	24,065
% within ethnic group	2.5	5.3	6.0	4.0	7.1	6.8	2.9
Adjusted residual	−56.5	47.4	24.0	4.6	12.1	9.7	
Private insurance/managed care							
Count	180,868_a_	24,849_b_	4224_b_	1178_a,b_	840_c_	601_c_	212,560
% within ethnic group	25.9	24.4	24.8	25.1	35.5	34.4	25.8
Adjusted residual	8.3	−10.5	−3.0	−1.1	10.9	8.3	
Medicaid							
Count	34,118_a_	14,083_b_	2380_b_	789_c_	291_b_	258_b,c_	51,919
% within ethnic group	4.9	13.8	13.9	16.8	12.3	14.8	6.3
Adjusted residual	−122.5	105.9	41.6	29.7	12.1	14.6	
Medicare							
Count	446,107_a_	53,889_b_	9025_b_	2401_b_	1008_c_	707_c_	513,137
% within ethnic group	63.9	52.9	52.9	51.1	42.6	40.4	62.2
Adjusted residual	77.5	−64.9	−25.2	−15.7	−19.6	−18.7	
Other government							
Count	9245_a_	1512_b_	101_c_	45_a,c_	21_a,b,c_	10_a,c_	10,934
% within ethnic group	1.3	1.5	0.6	1.0	0.9	0.6	1.3
Adjusted residual	0.1	4.8	−8.5	−2.2	−1.9	−2.8	
Insurance status unknown							
Count	10,207_a_	2129_b,c_	314_c_	94_b,c_	36_a,c_	53_b_	12,833
% within ethnic group	1.5	2.1	1.8	2.0	1.5	3.0	1.6
Adjusted esidual	−15.8	14.8	3.0	2.5	−0.1	5.0	

*WNH*, White non-Hispanic; *EA*, East Asian (Chinese, Japanese, Korean); *SEA*, Southeast Asian (Filipino, Vietnamese, Laotian, Hmong, Thai); *SA*, South Asian (Indian, Pakistani, Asian Indian/Pakistani not otherwise specified).

∗ Within a given table row, cells annotated with the same subscript letter indicate that the column proportions do not differ significantly from each other at the .05 level. Similarly, within a given table row, cells annotated with different subscript letters indicate that the column proportions do differ significantly from each other at the .05 level. For example, for the Insurance Status Unknown row: WNH is significantly different from Black, Hispanic, EA, and SA; Black is only significantly different from WNH; Hispanic is significantly different from WNH and SA; EA is only significantly different from WNH; SEA is only significantly different from SA; SA is significantly different from WNH, Hispanic, and SEA.

**Table 4:** Noncompletion of high school degree × ethnic group cross-tabulation

Noncompletion of high school degree	Ethnic group						Total
	WNH	Black	Hispanic	EA	SEA	SA	
First quartile∗ <6.3%							
Count	158,527_a_	6539_b_	1729_c_	1177_d_	538_a,d_	514_e_	169,024
% within ethnic group	23.0	6.5	10.2	25.2	22.9	29.7	20.7
Adjusted residual	118.1	−118.7	−34.0	7.6	2.6	9.2	
Fourth quartile∗ ≥17.6%							
Count	120,827_a_	42,309_b_	8131_c_	1522_d_	474_e_	361_e_	173,624
% within ethnic group	17.5	42.1	48.1	32.6	20.2	20.8	21.3
Adjusted residual	−194.2	172.2	86.1	18.9	−1.3	−0.4	

*WNH*, White non-Hispanic; *EA*, East Asian (Chinese, Japanese, Korean); *SEA*, Southeast Asian (Filipino, Vietnamese, Laotian, Hmong, Thai); *SA*, South Asian (Indian, Pakistani, Asian Indian/Pakistani not otherwise specified).

∗ Within a given table row, cells annotated with the same subscript letter indicate that the column proportions do not differ significantly from each other at the .05 level. Similarly, within a given table row, cells annotated with different subscript letters indicate that the column proportions do differ significantly from each other at the .05 level. For example, for the First Quartile row: WNH is significantly different from Black, Hispanic, EA, and SA; Black is significantly different from WNH, Hispanic, EA, SEA, and SA; Hispanic is significantly different from WNH, Black, EA, SEA, and SA; EA is significantly different from WNH, Black, Hispanic, and SA; SEA is significantly different from Black, Hispanic, and SA; SA is significantly different from WNH, Black, Hispanic, EA, and SEA.

### Clinical Stage

Table 5 depicts clinical stage group by ethnic group cross-tabulation employing our Asian American composite group, whereas Table 6 utilizes the 3 Asian American ethnic subgroups as the basis for comparison. In all clinical stages except clinical stage IV, Asian Americans were underrepresented relative to White non-Hispanics and additionally underrepresented to Blacks and Hispanics in clinical stages IIB and IIIA. In clinical stage IV, Asian Americans were overrepresented relative to White non-Hispanics, Blacks, and Hispanics (Asian Americans 51.0%, White non-Hispanics 42.2%, Blacks 47.5%, and Hispanics 47.0%) (Table 5). Further nuances are discovered when examining Asian American ethnic groups in Table 6. For example, Southeast Asians were underrepresented relative to East Asians and South Asians in clinical stage IA while being overrepresented relative to East Asians and South Asians in clinical stage IV (clinical stage IA: East Asians 19.8%, Southeast Asians 13.3%, and South Asians 17.4%; clinical stage IV: East Asians 47.2%, Southeast Asians 57.4%, and South Asians 52.2%) (Table 6).

**Table 5:** Clinical stage group × ethnic group cross-tabulation (combined Asian American group)

Clinical stage∗	Ethnic group				Total
	WNH	Black	Hispanic	AsAm	
IA					
Count	136,336_a_	14,518_b_	3008_c_	1550_c_	155,412
% within ethnic group	19.5	14.3	17.6	17.6	18.8
Adjusted residual	38.7	−39.8	−4.0	−3.0	
IB					
Count	58,555_a_	6842_b_	1231_b_	586_b_	67,214
% within ethnic group	8.4	6.7	7.2	6.7	8.1
Adjusted residual	19.3	−17.7	−4.5	−5.1	
IIA					
Count	23,066_a_	2889_b_	511_a, b_	243_b_	26,709
% within ethnic group	3.3	2.8	3.0	2.8	3.2
Adjusted residual	8.4	−7.7	−1.8	−2.5	
IIB					
Count	28,720_a_	3904_b_	608_b_	235_c_	33,467
% within ethnic group	4.1	3.8	3.6	2.7	4.1
Adjusted residual	6.6	−3.8	−3.3	−6.6	
IIIA					
Count	87,137_a_	13,354_b_	2034_a_	885_c_	103,410
% within ethnic group	12.5	13.1	11.9	10.0	12.5
Adjusted residual	−2.5	6.1	−2.4	−7.1	
IIIB					
Count	69,659_a_	11,940_b_	1655_a_	821_a_	84,065
% within ethnic group	10.0	11.7	9.7	9.3	10.2
Adjusted residual	−14.2	17.4	−2.1	−2.7	
IV					
Count	294,300_a_	48,366_b_	8016_b_	4489_c_	355,171
% within ethnic group	42.2	47.5	47.0	51.0	43.0
Adjusted residual	−36.5	30.8	10.5	15.1	

*WNH*, White non-Hispanic; *AsAm*, Asian American (East Asian: Chinese, Japanese, Korean; Southeast Asian: Filipino, Vietnamese, Laotian, Hmong, Thai; South Asian: Indian, Pakistani, Asian Indian/Pakistani not otherwise specified).

∗ Within a given table row, cells annotated with the same subscript letter indicate that the column proportions do not differ significantly from each other at the .05 level. Similarly, within a given table row, cells annotated with different subscript letters indicate that the column proportions do differ significantly from each other at the .05 level. For example, for the Clinical Stage IIA row: WNH is significantly different from Black and AsAm; Black is only significantly different from WNH; Hispanic is not significantly different from any group; AsAm is only significantly different from WNH.

**Table 6:** Clinical stage group × ethnic group cross-tabulation

Clinical stage∗	Ethnic group						Total
	WNH	Black	Hispanic	EA	SEA	SA	
IA							
Count	136,336_a_	14,518_b_	3008_c_	931_a_	315_b_	304_a,c_	155,412
% within ethnic group	19.5	14.3	17.6	19.8	13.3	17.4	18.8
Adjusted residual	38.7	−39.8	−4.0	1.7	−6.9	−1.5	
IB							
Count	58,555_a_	6842_b,c,d,e_	1231_d,e_	338_c,e_	125_b_	123_a,b,c,d,e_	67,214
% within ethnic group	8.4	6.7	7.2	7.2	5.3	7.0	8.1
Adjusted residual	19.3	−17.7	−4.5	−2.4	−5.1	−1.7	
IIA							
Count	23,066_a_	2889_b_	511_a,b_	128_a,b_	66_a,b_	49_a,b_	26,709
% within ethnic group	3.3	2.8	3.0	2.7	2.8	2.8	3.2
Adjusted residual	8.4	−7.7	−1.8	−2.0	−1.2	−1.0	
IIB							
Count	28,720_a_	3904_b_	608_b,c_	132_c_	58_c_	45_b,c_	33,467
% within ethnic group	4.1	3.8	3.6	2.8	2.5	2.6	4.1
Adjusted residual	6.6	−3.8	−3.3	−4.3	−4.0	−3.1	
IIIA							
Count	87,137_a_	13,354_b_	2034_a_	485_c_	219_c_	181_a,c_	103,410
% within ethnic group	12.5	13.1	11.9	10.3	9.3	10.4	12.5
Adjusted Residual	−2.5	6.1	−2.4	−4.6	−4.8	−2.7	
IIIB							
Count	69,659_a_	11,940_b_	1655_a,c_	465_a,c_	223_a,c_	133_c_	84,065
% within Ethnic Group	10.0	11.7	9.7	9.9	9.4	7.6	10.2
Adjusted Residual	−14.2	17.4	−2.1	−0.6	−1.2	−3.6	
IV							
Count	294,300_a_	48,366_b_	8016_b_	2218_b_	1358_c_	913_d_	355,171
% within ethnic group	42.2	47.5	47.0	47.2	57.4	52.2	43.0
Adjusted residual	−36.5	30.8	10.5	5.8	14.2	7.8	

*WNH*, White non-Hispanic; *EA*, East Asian (Chinese, Japanese, Korean); *SEA*, Southeast Asian (Filipino, Vietnamese, Laotian, Hmong, Thai); *SA*, South Asian (Indian, Pakistani, Asian Indian/Pakistani not otherwise specified).

∗ Within a given table row, cells annotated with the same subscript letter indicate that the column proportions do not differ significantly from each other at the .05 level. Similarly, within a given table row, cells annotated with different subscript letters indicate that the column proportions do differ significantly from each other at the .05 level. For example, for the Clinical Stage IB row: WNH is significantly different from Black, Hispanic, EA, and SEA; Black is only significantly different from WNH; Hispanic is significantly different from WNH and SEA; EA is significantly different from WNH and SEA; SEA is significantly different from WNH, Hispanic, and EA; SA is not significantly different from any group.

Table E1 provides a visual summary of over- and underrepresentation of the Asian American composite group as well as subgroups. Asian Americans were underrepresented relative to White non-Hispanics in all stages except clinical stage IIIB (no difference) and IV (overrepresented), reflecting the trend seen in the subgroups. The converse was largely true for clinical stage IV, but East Asians were underrepresented relative to Southeast Asians and South Asians in clinical stage IV. Asian Americans were underrepresented relative to White non-Hispanics in clinical stage IIA, yet no disaggregated subgroup in isolation was identified to be over- or underrepresented.

### Survival

Table 7 depicts a cross-tabulation of vital sign status alive at 30 days or date of last contact by clinical stage by ethnic group employing our Asian American composite group, whereas Table 8 utilizes the 3 Asian American ethnic subgroups as the basis for comparison. In all clinical stages, Asian American survival was overrepresented relative to White non-Hispanics, Blacks, and Hispanics except in clinical stage IIA and IIB where Asian American survival was overrepresented relative to White non-Hispanics and Blacks (Table 7). However, when comparing the subgroups in Table 8, it was found that Southeast Asian and South Asian survival were not significantly different than White non-Hispanics or Blacks in clinical stage IIA and IIB.

**Table 7:** Vital status at date of last contact × ethnic group × clinical stage group cross-tabulation (combined Asian American group)

Clinical stage∗	Vital status at date of last contact		Ethnic group				Total
			WNH	Black	Hispanic	AsAm	
IA	Alive	Count	62,673_a_	6834_b_	1743_c_	1044_d_	72,294
		% within ethnic group	52.6	54.0	68.7	79.2	53.3
		Adjusted residual	−13.7	1.7	15.7	18.9	
IB	Alive	Count	19,814_a_	2401_a_	582_b_	320_c_	23,117
		% within ethnic group	37.0	38.4	53.1	61.2	37.6
		Adjusted residual	−8.5	1.4	10.7	11.2	
IIA	Alive	Count	7851_a_	1019_a_	234_b_	114_b_	9218
		% within ethnic group	38.6	40.2	53.1	57.0	39.2
		Adjusted residual	−4.8	1.1	6.0	5.2	
IIB	Alive	Count	7149_a_	1022_a_	242_b_	97_b_	8510
		% within ethnic group	27.3	28.8	42.5	46.2	27.9
		Adjusted residual	−5.6	1.3	7.8	5.9	
IIIA	Alive	Count	16,737_a_	2864_b_	604_c_	315_d_	20,520
		% within ethnic group	21.2	23.8	33.2	39.7	21.9
		Adjusted residual	−12.6	5.5	11.8	12.1	
IIIB	Alive	Count	7505_a_	1553_b_	339_c_	214_d_	9611
		% within ethnic group	11.4	13.8	21.6	28.2	12.1
		Adjusted residual	−13.3	6.0	11.6	13.7	
IV	Alive	Count	19,560_a_	3884_b_	1367_c_	937_d_	25,748
		% within ethnic group	7.3	8.8	19.0	23.6	8.0
		Adjusted residual	−31.2	7.3	35.1	36.7	

*WNH*, White non-Hispanic; *AsAm*, Asian American (East Asian: Chinese, Japanese, Korean; Southeast Asian: Filipino, Vietnamese, Laotian, Hmong, Thai; South Asian: Indian Pakistani, Asian Indian/Pakistani not otherwise specified).

∗ Within a given table row, cells annotated with the same subscript letter indicate that the column proportions do not differ significantly from each other at the .05 level. Similarly, within a given table row, cells annotated with different subscript letters indicate that the column proportions do differ significantly from each other at the .05 level. For example, for the Clinical Stage IB row: WNH is significantly different from Hispanic and AsAm; Black is significantly different from Hispanic and AsAm; Hispanic is significiantly different from WNH, Black, and AsAm; AsAm is significantly different from WNH, Black, and Hispanic.

**Table 8:** Vital status at date of last contact × ethnic group × clinical stage group cross-tabulation

Clinical stage∗	Vital status at date of last contact		WNH	Ethnic group					Total
				Black	Hispanic	EA	SEA	SA	
IA	Alive	Count	62,673_a_	6834_b_	1743_c_	639_d_	204_c,d_	201_c,d_	72,294
		% within ethnic group	52.6	54.0	68.7	81.2	76.1	76.1	53.3
		Adjusted residual	−13.7	1.7	15.7	15.7	7.5	7.5	
IB	Alive	Count	19,814_a_	2401_a,b_	582_c_	199_d_	63_c,d_	58_b,c,d_	23,117
		% within ethnic group	37.0	38.4	53.1	65.7	58.3	51.8	37.6
		Adjusted residual	−8.5	1.4	10.7	10.1	4.4	3.1	
IIA	Alive	Count	7851_a_	1019_a_	234_b_	68_b_	25_a,b_	21_a,b_	9218
		% within ethnic group	38.6	40.2	53.1	63.6	44.6	56.8	39.2
		Adjusted residual	−4.8	1.1	6.0	5.2	0.8	2.2	
IIB	Alive	Count	7149_a_	1022_a_	242_b_	62_b_	19_a, b_	16_a,b_	8510
		% within ethnic group	27.3	28.8	42.5	52.1	37.3	40.0	27.9
		Adjusted residual	−5.6	1.3	7.8	5.9	1.5	1.7	
IIIA	Alive	Count	16,737_a_	2864_b_	604_c_	177_c_	76_c_	62_c_	20,520
		% within ethnic group	21.2	23.8	33.2	40.3	39.8	37.8	21.9
		Adjusted residual	−12.6	5.5	11.8	9.3	6.0	4.9	
IIIB	Alive	Count	7505_a_	1553_b_	339_c_	132_d_	42_b,c,d_	40_d_	9611
		% within ethnic group	11.4	13.8	21.6	30.6	19.9	34.2	12.1
		Adjusted residual	−13.3	6.0	11.6	11.8	3.5	7.3	
IV	Alive	Count	19,560_a_	3884_b_	1367_c_	503_d_	219_c_	215_d_	25,748
		% within ethnic group	7.3	8.8	19.0	26.0	17.9	26.6	8.0
		Adjusted residual	−31.2	7.3	35.1	29.4	12.9	19.6	

*WNH*, White non-Hispanic; *EA*, East Asian (Chinese, Japanese, Korean); *SEA*, Southeast Asian (Filipino, Vietnamese, Laotian, Hmong, Thai); *SA*, South Asian (Indian, Pakistani, Asian Indian/Pakistani not otherwise specified).

∗ Within a given table row, cells annotated with the same subscript letter indicate that the column proportions do not differ significantly from each other at the .05 level. Similarly, within a given table row, cells annotated with different subscript letters indicate that the column proportions do differ significantly from each other at the .05 level. For example, for the Clinical Stage IB row: WNH is significantly different form Hispanic, EA, SEA, and SA; Black is significantly different from Hispanic, EA, and SEA; Hispanic is significantly different from WNH, Black, and EA; EA is significantly different from WNH, Black, and Hispanic; SEA is significantly different from WNH and Black; SA is only significantly different from WNH.

Table E2 provides a visual summary of over- and underrepresentation of the Asian American composite group as well as subgroups regarding survival. Asian American survival was consistently overrepresented in all stages relative to White non-Hispanics and Blacks, but within the subgroups, several nuances became apparent. For example, all subgroups’ survival was equivalent in clinical stage IA or IIIB, but Southeast Asian survival in clinical stage IV was significantly lower than in East Asians and South Asians. Such differences were obscured when the comparison group is broad (ie, the pooled Asian American group).

## Discussion

**Figure 1:**
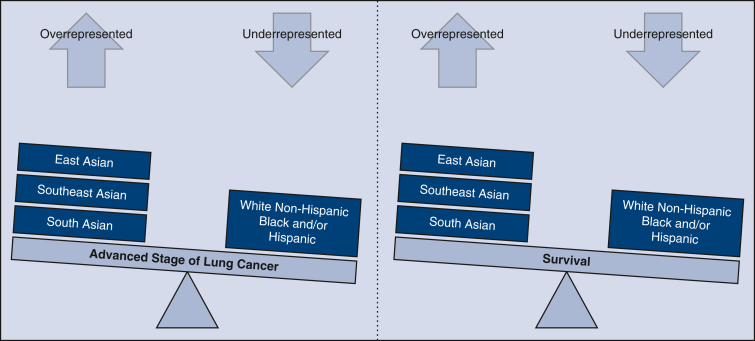
Comparison of advanced stage lung cancer and survival between ethnic groups. East Asian, Southeast Asian, and South Asian individuals are more likely to be diagnosed at advanced stages of lung cancer yet demonstrate higher survival compared with White Non-Hispanic, Black, and/or Hispanic with differences between subgroups.

**Figure 2:**
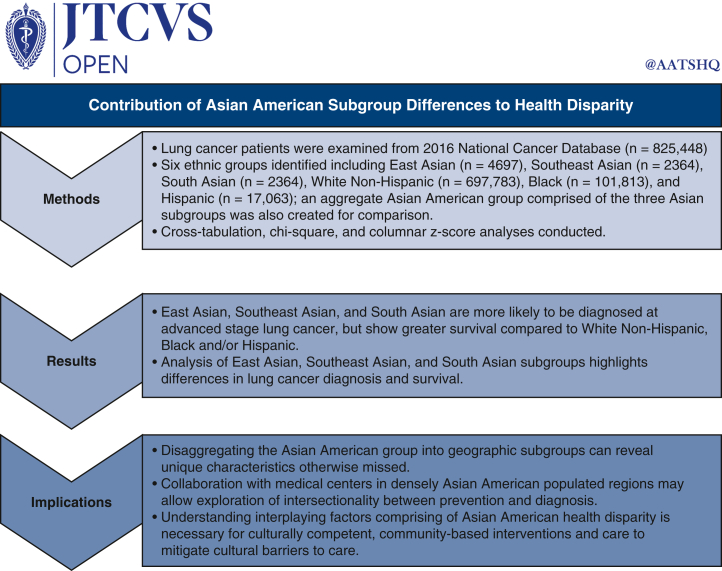
Graphical abstract of the study. Graphic outlines Asian American health disparities in lung cancer with a novel analytic approach.

The NCDB collects data on patients with any cancer diagnosis from more than 1500 cancer-accredited facilities. Despite this large source of data, analysis of Asian Americans with lung cancer has been difficult in the past when compared with other major US ethnic groups due to low counts within ethnic subgroups. By applying columnar *z*-score analysis with adjusted standardized residuals, we attempted to overcome this issue, which to our knowledge was the first time this analytical approach has been applied to this type of study. We employed the terms *underrepresented* and *overrepresented* when cross-tabulation percentages were accompanied by a *z*-score proportion difference of *P* < .05 (Figure 1). In addition, our analysis went a step further than prior Asian American lung cancer studies by looking at clinical stage at diagnosis and survival in East Asian, Southeast Asian, and South Asian ethnic subgroups (see Figure 2 for a graphical abstract of the study). The results were consistent with other studies that examined lung cancer stage and survival in Asian Americans,^17^, ^18^, ^19^ except that we also examined Asian American subgroups.

Among the biggest obstacles in studying Asian American health disparities is the issue of aggregate data. The generalization of Asian Americans as 1 group can mask the unique disease risk of different subgroups and assume similar disease risk as the White non-Hispanic majority.^10^ Tables E1 and E2 demonstrate the underlying issues and limitation of aggregating Asian Americans as 1 group. Asian Americans demonstrated significantly higher proportion of clinical stage IV lung cancer diagnosis compared with White non-Hispanics, Blacks, and Hispanics. However, when examining the Asian American subgroups, clinical stage IV diagnosis was significantly higher in Southeast Asians than East Asians and South Asians and significantly higher in South Asians than East Asians (Table E1). With disaggregated data, physicians may assume that all Asian American patients have a similar degree of being diagnosed at advanced stages of lung cancer, which can in turn negatively influence the care of Asian American patients. Additionally, Asian American survival was significantly higher in all stages of lung cancer compared with White non-Hispanics and Blacks, but Southeast Asians’ survival in clinical stage IV was significantly lower than East Asians and South Asians (Table E2). Without the knowledge of this difference between subgroups, Southeast Asian patients may be treated similarly as East Asian and South Asian patients, which could potentially result in unexpected outcomes.

The interplay of factors associated with the complex findings of advanced diagnosis yet greater survival with differences between subgroups includes cultural beliefs, environmental exposures, genetics, immigration, diet, and barriers to care, just to name a few, which may begin to unlock clues to understanding Asian American health disparities.

Because lung cancer is the leading cause of death for Asian Americans, smoking is a significant risk factor and the number-1 underlying cause of lung cancer worldwide.^20^ Smoking prevalence ranged from 7.6% among Chinese and Indians to 20.0% among Koreans.^21^ When smoking cessation in Asian Americans was examined, only 34.2% of Asian Americans who smoke were advised to quit compared with 60.2% White non-Hispanics and 42.2% Hispanics who smoke. Additionally, Asian Americans who smoke received less counseling and/or medication for smoking cessation compared with White non-Hispanics who smoke (20.5% vs 34.3%).^22^ The discrepancy in smoking prevalence and cessation in Asian Americans could be a direct result of aggregating Asian Americans as 1 group, which demonstrates the importance of examining Asian American subgroups. Given the high prevalence of smoking in Asian cultures, environmental tobacco smoke is a factor to consider as tobacco use by spouse was associated with a 30% excess risk of lung cancer.^23^ Other environmental risk factors include air pollution and cooking oil fumes.^24^^,^^25^ Indoor air pollutants from Chinese-style cooking has been linked to increased risk of lung cancer.^25^ Lastly, dietary factors may be protective, such as high consumption of vegetables, fruits, and soy products in Asian cultures.^26^

The potential of lung cancer screening has also poorly penetrated Asian Americans because they had a lower rate of cancer screening than White non-Hispanics and was the only racial group where screening disparity was not well explained by socioeconomic factors.^27^ This is possibly due to lack of knowledge of the health care system, language barriers, and culture. Many Asian cultures believe illness as one's fate, so preventative health care is not a widely embraced practice.^11^ Another contributor to potentially explain delayed diagnosis could stem from the labeling of Asian Americans as the so-called model minority. By accepting this stereotype in attempt to assimilate, Asian Americans may be reluctant to disclose and advocate for their health.^28^ These stereotypes may lead to the perception that Asian Americans do not need help. In fact, Asian Americans were the least likely among all racial groups to have seen a physician in the past 12 months.^12^

Further complicating these issues is that not every aforementioned factor can or should be generalized toward all Asian Americans because some Asian American ethnic groups immigrated to the United States more recently than others. Many Japanese immigrants arrived in the 19th century with 63% of Japanese Americans having been in the United States for more than 10 years.^13^ Depending on the timing of immigration, Asian Americans may carry different risk factors and retain different amounts of cultural beliefs. Because of the heterogeneity of Asian Americans, they should not be regarded or treated similarly. Thus, culturally competent care is crucial for Asian American patients.

Studies have shown improved survival in Asian Americans with NSCLC compared with White non-Hispanics, which is consistent with our findings.^29^, ^30^, ^31^ This perhaps is due to the role of epidermal growth factor receptor (EGFR) mutations and polymorphism.^29^^,^^32^, ^33^, ^34^ Persons who do not smoke have more EGFR mutations and improved survival.^29^ It has been well established that there is a predominance of Asian American patients who do not smoke with NSCLC, especially Asian American women.^29^^,^^35^ A high level of EGFR protein was also linked to poorer outcome in NSCLC.^32^ Compared with other ethnic groups, Asian Americans had lower EGFR expression due to polymorphism.^33^ Along with the higher frequency of amplification in a closely related *HER2* gene in East Asians, one can speculate the influence of genetic diversity on clinical outcomes such as survival.^34^

### Limitations

There are several limitations to this study. First, a total of 11 out of 18 Asian ethnicities were divided based on geographic origins into 3 Asian American subgroups in this study due to limited number of patients in each Asian ethnicity. Our creation of cohorts resulted in the exclusion of 5 mixed Asian ethnicities within the NCDB. This was done mostly due to American cultural influence within the area (eg, American Samoa and Philippines). Secondly, histological subtypes of lung cancer were not explored in this study due to the uncertainty of its clinical significance given the relatively small number of patients in each Asian American subgroup. With consistent and comprehensive identity collection nationwide, further investigation is possible and warranted to aid in understanding of these results. Lastly, because screening and smoking status are crucial components of lung cancer risk reduction, collaboration with medical centers in densely Asian American-populated regions may allow the exploration of intersectionality between risk reduction and diagnosis, which we were unable to do with the NCDB data. This may allow future studies to distinguish more specific characteristics within the Asian American subpopulations.

## Conclusions

This study demonstrated the importance of disaggregating Asian Americans as 1 group while also comparing them to the dominant US populations allowing us to both quantify and analyze these differences. Although Asian Americans were more likely to be diagnosed at advanced stages of lung cancer, they demonstrated higher survival compared with White non-Hispanics, Blacks, and/or Hispanics, whereas differences between Asian American subgroups were also apparent. Disaggregating the Asian American umbrella group can reveal unique characteristics of individual subgroups otherwise missed. Further investigation to explore differences between the Asian American subgroups is necessary to make definitive conclusions. Understanding the interplaying factors comprising Asian American health disparities is recommended for culturally competent care, community-based interventions, and personalized medicine.

### Webcast

You can watch a Webcast of this AATS meeting presentation by going to: https://www.aats.org/resources/identifying-asian-american-lung-cancer-disparities-a-novel-analytic-approach.

**Figure:**
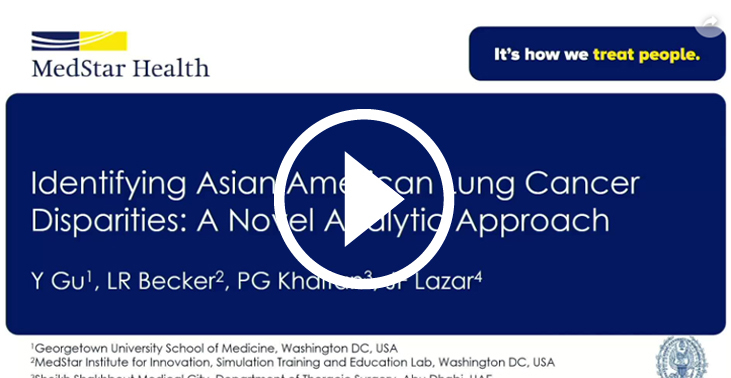

## Conflict of Interest Statement

The authors reported no conflicts of interest.

The *Journal* policy requires editors and reviewers to disclose conflicts of interest and to decline handling manuscripts for which they may have a conflict of interest. The editors and reviewers of this article have no conflicts of interest.

## Figures and Tables

**Table E1:** Summary of under- and overrepresentation of Asian subgroups in lung cancer clinical stages

Clinical stage	Asian group																				
Comparison group	EA						SEA						SA						AsAm		
	WNH	Black	Hispanic	EA	SEA	SA	WNH	Black	Hispanic	EA	SEA	SA	WNH	Black	Hispanic	EA	SEA	SA	WNH	Black	Hispanic
Stage IA	–	–	–		–	–	↓	–	↓	↓		↓	–	–	–	–	–		↓	↑	–
Stage IB	↓	–	–		↑	–	↓	–	↓	↓		–	–	–	–	–	–		↓	–	–
Stage IIA	–	–	–		–	–	–	–	–	–		–	–	–	–	–	–		↓	–	–
Stage IIB	↓	↓	–		–	–	↓	↓	–	–		–	↓	–	–	–	–		↓	↓	↓
Stage IIIA	↓	↓	↓		–	–	↓	↓	↓	–		–	–	↓	–	–	–		↓	↓	↓
Stage IIIB	–	–	–		–	–	–	–	–	–		–	↓	↓	–	–	–		–	↓	–
Stage IV	↑	–	–		↓	↓	↑	↑	↑	↑		↑	↑	↑	↑	↑	↓		↑	↑	↑

Upward ↑ and downward ↓ facing arrows indicate over- and underrepresentation relative to comparison groups at *P* < .05, respectively. A dash indicates no significant difference in representation. *EA*, East Asian (Chinese, Japanese, Korean); *SEA*, Southeast Asian (Filipino, Vietnamese, Laotian, Hmong, Thai); *SA*, South Asian (Indian, Pakistani, Asian Indian/Pakistani not otherwise specified); *AsAm*, Asian American (East Asian, Southeast Asian, South Asian); *WNH*, White non-Hispanic.

**Table E2:** Summary of under- and overrepresentation of Asian subgroups in vital status = alive at date of last contact

Vital status at date of last contact = Alive	Asian group																				
	EA						SEA						SA						AsAm		
Comparison group	WNH	Black	Hispanic	EA	SEA	SA	WNH	Black	Hispanic	EA	SEA	SA	WNH	Black	Hispanic	EA	SEA	SA	WNH	Black	Hispanic
Stage IA	↑	↑	↑		–	–	↑	↑	–	–		–	↑	↑	–	–	–		↑	↑	↑
Stage IB	↑	↑	↑		–	–	↑	↑	–	–		–	↑	–	–	–	–		↑	↑	↑
Stage IIA	↑	↑	–		–	–	–	–	–	–		–	–	–	–	–	–		↑	↑	–
Stage IIB	↑	↑	–		–	–	–	–	–	–		–	–	–	–	–	–		↑	↑	–
Stage IIIA	↑	↑	–		–	–	↑	↑	–	–		–	↑	↑	–	–	–		↑	↑	↑
Stage IIIB	↑	↑	↑		–	–	↑	–	–	–		–	↑	↑	↑	–	–		↑	↑	↑
Stage IV	↑	↑	↑		↑	-	↑	↑	-	↓		↓	↑	↑	↑	-	↑		↑	↑	↑

Upward ↑ and downward ↓ facing arrows indicate over- and underrepresentation relative to comparison groups at *P* < .05, respectively. A dash indicates no significant difference in representation. *EA*, East Asian (Chinese, Japanese, Korean); *SEA*, Southeast Asian (Filipino, Vietnamese, Laotian, Hmong, Thai); *SA*, South Asian (Indian, Pakistani, Asian Indian/Pakistani not otherwise specified); *AsAm*, Asian American (East Asian, Southeast Asian, South Asian); *WNH*, White non-Hispanic.
